# Comprehensive computational analysis of Hmd enzymes and paralogs in methanogenic Archaea

**DOI:** 10.1186/1471-2148-9-199

**Published:** 2009-08-11

**Authors:** Aaron D Goldman, John A Leigh, Ram Samudrala

**Affiliations:** 1 Department of Microbiology, University of Washington, Seattle, WA, USA; 2 NSF IGERT Program in Astrobiology, University of Washington, Seattle, WA, USA

## Abstract

**Background:**

Methanogenesis is the sole means of energy production in methanogenic Archaea. H_2_-forming methylenetetrahydromethanopterin dehydrogenase (Hmd) catalyzes a step in the hydrogenotrophic methanogenesis pathway in class I methanogens. At least one *hmd *paralog has been identified in nine of the eleven complete genome sequences of class I hydrogenotrophic methanogens. The products of these paralog genes have thus far eluded any detailed functional characterization.

**Results:**

Here we present a thorough computational analysis of Hmd enzymes and paralogs that includes state of the art phylogenetic inference, structure prediction, and functional site prediction techniques. We determine that the Hmd enzymes are phylogenetically distinct from Hmd paralogs but share a common overall structure. We predict that the active site of the Hmd enzyme is conserved as a functional site in Hmd paralogs and use this observation to propose possible molecular functions of the paralog that are consistent with previous experimental evidence. We also identify an uncharacterized site in the N-terminal domains of both proteins that is predicted by our methods to directly impart function.

**Conclusion:**

This study contributes to our understanding of the evolutionary history, structural conservation, and functional roles, of the Hmd enzymes and paralogs. The results of our phylogenetic and structural analysis constitute datasets that will aid in the future study of the Hmd protein family. Our functional site predictions generate several testable hypotheses that will guide further experimental characterization of the Hmd paralog. This work also represents a novel approach to protein function prediction in which multiple computational methods are integrated to achieve a detailed characterization of proteins that are not well understood.

## Background

The methanogens are a diverse, but phylogenetically related, group of Archaea. Methanogenic Archaea have been isolated from habitats ranging from mammalian gut flora to deep sea hydrothermal vents. Methanogens are comprised of two taxonomic classes known as class I and class II [1-3]. Class I methanogens include the orders *Methanococcales*, *Methanobacteriales*, and *Methanopyrales*, while class II methanogens include the orders *Methanosarcinales *and *Methanomicrobiales*.

**Figure 1:**
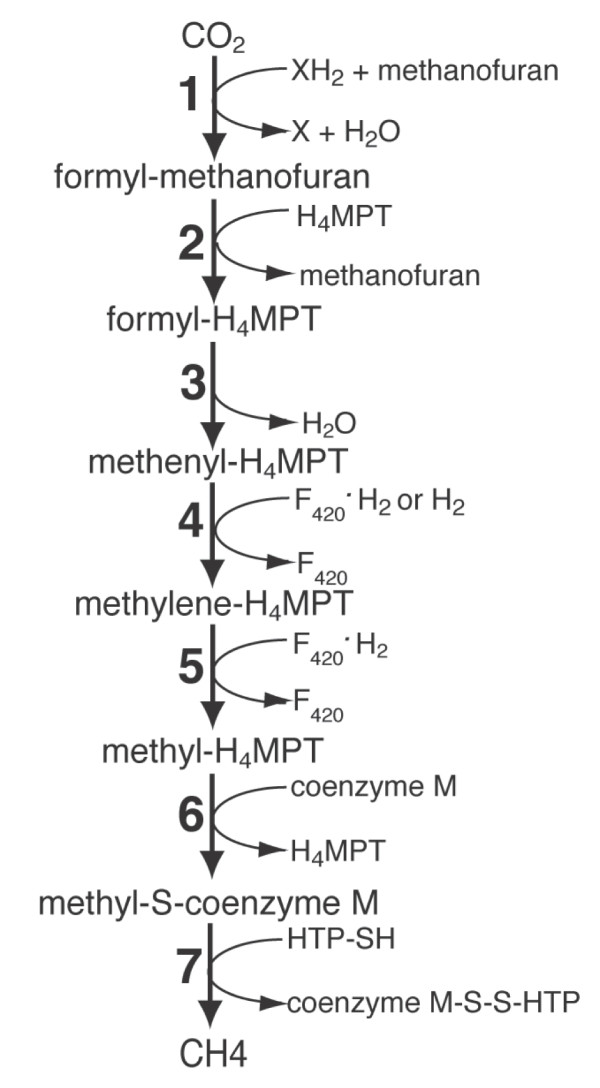
**The hydrogenotrophic methanogenesis pathway of class I methanogens**. The pathway diagram was adapted from [5] with permission from the author and publisher. The fourth step of this pathway can be catalyzed by either Hmd, which uses H_2 _as an electron donor, or Mtd, which uses F_420_^•^H_2 _as an electron donor. H_4_MPT = tetrahydromethanopterin, F_420 _= coenzyme F_420_, and HTP = S-S-7-mercaptoheptanoylthreonine phosphate.

The three known methanogenesis pathways are distinguished with regards to the electron source. These are hydrogenotrophic methanogenesis, acetoclastic methanogenesis, and methylotrophic methanogenesis [4]. Hydrogenotrophic methanogenesis involves the reduction of CO_2 _to CH_4_, utilizing H_2 _and reduced cofactors as electron donors through a seven step pathway (Figure 1). Many hydrogenotrophic methanogens are autotrophic, requiring only CO_2_, H_2_, and inorganic salts to produce energy through methanogenesis and synthesize biomass through CO_2 _fixation [5].

The fourth step in the hydrogenotrophic methanogenesis of class I methanogens involves the reduction of N^5^,N^10^-methenyltetrahydromethanopterin (methenyl-H_4_MPT) to N^5^,N^10^-methylene-H_4_MPT. Class II methanogens differ in their use of methanosarcinapterin rather than H_4_MPT as the C_1 _carrier. This step in class I methanogens can be carried out by either of two different enzymes. Coenzyme F_420_-dependent methylene-H_4_MPT dehydrogenase (Mtd) reduces methenyl-H_4_MPT using reduced coenzyme F_420 _as the electron donor. H_2_-forming methylene-H_4_MPT dehydrogenase (Hmd) reduces methenyl-H_4_MPT to methylene-H_4_MPT using H_2 _as an electron source. Afting et al. [6] observed in *Methanothermobacter marbugensis *that Hmd has a specific activity greater than that of Mtd under nickel-limited, ammonia-limited, and non-limited conditions while Mtd has a specific activity greater than that of Hmd under hydrogen-limited conditions. Hendrickson et al. [7] observed in *Methanococcus maripaludis *that *hmd *is upregulated proportional to growth rate and *mtd *is upregulated under hydrogen limitation.

The Hmd holoenzyme is comprised of a homodimer of 38 kDa subunits, two pyridone derivative cofactor molecules, and two iron atoms [8]. Each iron atom coordinates the reduction of methenyl-H_4_MPT and oxidation of H_2 _while bound to both Hmd and a cofactor molecule [8,9]. The apoenzyme of Hmd is stable and can be restored to active holoenzyme by the addition of cofactor [9]. Hmd is the only known hydrogenase that lacks an iron-sulfur cluster and is sometimes referred to as the 'iron-sulfur cluster-free hydrogenase'.

Almost all genomes of class I hydrogenotrophic methanogens contain both an *hmd *enzyme gene and at least one *hmd *paralog gene. Several species have two copies of the *hmd *paralog (referred to in this manuscript with arbitrary numeration as paralog_1 _and paralog_2_; see Additional file 1). Afting et al. [6] first showed in *M. marburgensis *that the protein products of *hmd *paralogs are present in the cell. Their study also revealed that Hmd paralog_1 _is detectable at low H_2_, while Hmd paralog_2 _is detectable at high H_2 _and that neither paralog show any observable hydrogenase activity. Recent unpublished work mentioned in a review by Shima and Thauer [10] indicates that Hmd paralog_1 _from *Methanocaldococcus jannaschii *can competitively bind cofactor and inhibit the activation of Hmd apoenzyme. Curiously, Hmd paralog_1 _in *M. jannaschii *was shown by Lipman et al. [11] to specifically bind prolyl-tRNA synthetase. While these results taken together constitute a partial characterization of Hmd paralogs, our understanding of these proteins and their role in methanogenesis is far from complete.

Here we present advanced computational analyses of Hmd enzymes and their paralogs from the genomes of sixteen class I hydrogenotrophic methanogens. The relationship of *hmd *enzyme and paralog sequences is demonstrated through phylogenetic analysis. The tertiary structures of Hmd enzymes and paralogs from five representative species are predicted using the top ranking modeling server of the last two CASP competitions [[12]; ]. Functional characterization of the Hmd paralogs is performed using a state of the art method recently developed by our group [13]. Taken together, these analyses form a thorough computational characterization of the Hmd enzymes and paralogs and generate several testable hypotheses regarding the molecular functions of both Hmd enzymes and paralogs.

## Results and discussion

### Sequence analysis

An exhaustive search for *hmd *genes was performed using PSI-BLAST [14] and the MetaCyc multi-genome browser [15]. This process identified thirty *hmd *enzyme and paralog sequences from sixteen species and strains of class I hydrogenotrophic methanogens. Several methanogen prephenate dehydrogenase genes were also identified by our search. We use these genes as a phylogenetic outgroup in the subsequent analysis. Complete genome sequences are available for eleven of the sixteen species and strains. Of these eleven, only the genomes of *Methanocorpusculum labreanum *and *Methanobrevibacter smithii *contain an *hmd *enzyme but not an *hmd *paralog. All *Methanococcus *spp. have only one *hmd *paralog gene, while *Methanocaldococcus jannaschii*, *Methanothermobacter marburgensis*, *Methanothermobacter thermautotrophicus*, and *Methanopyrus kandleri *have two *hmd *paralog genes. No species was found to have an *hmd *paralog, but not an *hmd *enzyme. Features of these genes, their GenInfo Identifiers, and their associated references [[16-23]; Copeland *et al.*, unpublished data; Hartmann and Thauer, *direct submission to NCBI databases *1996] are presented in Additional file 1. A ClustalW2 alignment of the protein sequences of these genes is included as Additional file 2.

### Phylogenetic analysis

**Figure 2:**
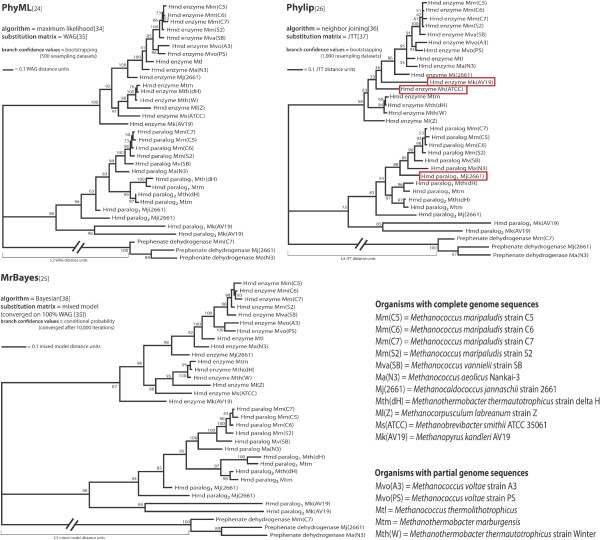
**Three phylogenetic trees of the Hmd protein family**. Each phylogeny was calculated independently using different software, tree calculation algorithms, and amino acid substitution models, which are displayed to the upper left of each tree. Archaeal prephenate dehydrogenase is used as an outgroup in each tree. The trees have differing branch lengths, but almost identical topologies. The Phylip tree differs from the other two at three leaf nodes highlighted in red. In these trees, Hmd enzymes and paralogs form two distinct monophyletic groups. Only two species with complete genome sequences, *M. smithii *and *M. labraenum*, have an Hmd enzyme and do not have an Hmd paralog. The phylogenies indicate that three independent duplications of the Hmd paralog took place in the lineage leading to *M. jannaschii*, the lineage leading to *M. kandleri*, and the lineage of the last common ancestor of *M. marburgensis *and *M. thermoauotrophicum*. This analysis suggests that Hmd paralogs have a conserved functional role in class I hydrogenotrophic methanogens.

Phylogenetic analysis of the thirty Hmd enzyme and paralog sequences was performed by three independent methods. In each tree, the three prephenate dehydrogenase sequences were used as an outgroup. Figure 2 shows the three trees and specifies the software, calculation algorithm, amino acid substitution matrix, and confidence score calculation method used to generate them. Though branch lengths differ between trees, the overall topology is identical between the PhyML [24] and MrBayes [25] trees and differs in only three terminal nodes of the Phylip [26] tree.

In all three trees, Hmd enzymes and paralogs form two distinct monophyletic groups. Curiously, the Hmd enzyme and paralog subtrees are considerably dissimilar regarding the placement of *M. jannaschii *sequences. These sequences are more basal in the paralog subtree than the enzyme subtree (with the exception of Hmd paralog_1 _in the Phylip tree). Bifurcation patterns in the tree suggest that paralog duplication has taken place independently in the lineages leading to *M. jannaschii*, *M. kandleri*, and the last common ancestor of *M. marburgensis *and *M. thermautotrophicus*. The two Hmd paralogs of *M. jannaschii *are paraphyletic in the PhyML and MrBayes trees and polyphyletic in the Phylip tree. The paralog duplicates of *M. kandleri *and the last common ancestor of *M. marburgensis *and *M. thermautotrophicus *both produce monophyletic topologies. It should be noted that *M. marburgensis *and *M. thermautotrophicus *were considered strains of a single species until recently [21].

These trees do not provide a conclusive explanation for the lack of a paralog sequence in *M. labraenum *or *M. smithii*. *M. labraenum *and *M. smithii *enzyme sequences are not basally branching, but were inherited from the last common ancestor of these species and the *Methanothermobacter *genus. Given that the *M. kandleri *paralog sequences appear in a subtree with the other paralog sequences, rather than branching from the base of the tree, it is likely that both *M. labraenum *and *M. smithii *lost the Hmd paralog late in evolution. It is therefore probable, but not certain, that the last common ancestor of all class I methanogens had both an Hmd enzyme and paralog.

### Structure modeling

Tertiary structure models of fourteen representative Hmd enzymes and paralogs were generated with I-TASSER [27,28], which was the best performing structure modeling server in the two most recent CASP competitions [[12], ]. The I-TASSER algorithm is an advanced modeling method that searches the SCOP database [29] for parent template structures, uses these parent structures to comparatively model short segments of the query protein, and connects these segments using *de novo *modeling techniques. Because the modeling is not dependent on comparison to a single homolog, this method can be considered a form of *de novo *structure modeling.

**Figure 3:**
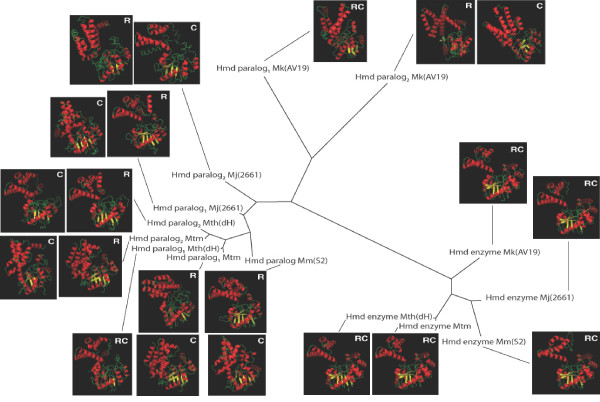
**Tertiary structure models of Hmd enzymes and paralogs superimposed onto a phylogenetic tree**. Models labeled "R" represent the model with the best RAPDF score [30] for a given protein. Models labeled "C" represent the model with the best C-score [27] for a given protein. Models labeled "RC" had both the best RAPDF and C-score for a given protein. Features of these models are summarized in Table 1. All models contain the same overall structure consisting of an N-terminal domain composed of α-helices and β-sheets and a C-terminal domain composed of α-helices. In the Hmd enzyme, the N-terminal domain contains the catalytic site while the C-terminal domain facilitates dimerization in the Hmd holoenzyme. In general, the N-terminal domain exhibits more structural variability than the C-terminal domain (see Table 1).

The structure of the Hmd enzyme from *M. jannaschii *has previously been solved by X-ray diffraction [[8]; PDB ID = 2b0j]. This structure was the most often used parent template of the top C-scoring [27] model of each protein. The next three most often used parent structures were dehydrogenases. These parent structures were arogenate dehydrogenase from *Synechocystis sp.*, hydroxyisobutyrate dehydrogenase from *Homo sapiens*, and prephenate dehydrogenase from *Aquifex aeolicus*. The resulting I-TASSER models were evaluated by both the C-score [27] and residue-specific all-atom probability discriminatory function (RAPDF) [30] scoring functions. These scoring functions measure the relative accuracy of a given model compared to other models of the same protein. C-score is determined by clustering the thousands of intermediate models generated during the I-TASSER run. Structures in the center of the largest clusters are assumed to be the most accurate. RAPDF determines the quality of a model by calculating the sum of logodds scores for all interatomic distances within the model derived from frequencies observed in diffraction structures. The model with the highest C-score also had the best RAPDF score in the case of all five Hmd enzymes and two of the nine Hmd paralogs. Figure 3 shows all top C-scoring and RAPDF-scoring models mapped onto a PhyML [24] phylogeny of the corresponding sequences. A summary of features of these models is given in Table 1. A concatenated file of all top C-scoring and RAPDF-scoring models in PDB format is available as Additional file 3.

**Table 1:** Features of Hmd enzyme and paralog structure models

**Model**	**RAPDF rank**	**C-score rank**	**Whole protein RMSD**	**N-terminal RMSD**	**C-terminal RMSD**
Enzyme_RC _Mm(S2)	1 of 5	1 of 5	5.5	4.4	18.8
Enzyme_RC _Mj(2661)	1 of 5	1 of 5	2.2	1.9	2.1
Enzyme_RC _Mth(dH)	1 of 5	1 of 5	11.2	9.7	11.6
Enzyme_RC _Mtm	1 of 5	1 of 5	10.6	9.2	7.1
Enzyme_RC _Mk(AV19)	1 of 5	1 of 5	16.1	15.3	7.2
Paralog_R _Mm(S2)	1 of 5	2 of 5	21.1	17.9	8.4
Paralog_C _Mm(S2)	4 of 5	1 of 5	20.5	17.4	16.7
Paralog_1-R _Mj(2661)	1 of 5	2 of 5	20.5	17.5	11.4
Paralog_1-C _Mj(2661)	3 of 5	1 of 5	21.4	17.5	10.1
Paralog_2-R _Mj(2661)	1 of 5	2 of 5	20.4	17.8	7.5
Paralog_2-C _Mj(2661)	3 of 5	1 of 5	20.7	17.8	16.4
Paralog_1-R _Mth(dH)	1 of 5	3 of 5	20.5	18.1	8.1
Paralog_1-C _Mth(dH)	2 of 5	1 of 5	19.9	17.8	10.0
Paralog_1-RC _Mth(dH)	1 of 5	1 of 5	20.6	17.7	11.1
Paralog_1-R _Mtm	1 of 5	5 of 5	21.6	17.9	15.4
Paralog_1-C _Mtm	5 of 5	1 of 5	20.7	17.4	15.8
Paralog_2-R _Mtm	1 of 5	5 of 5	21.1	17.6	11.1
Paralog_2-C _Mtm	3 of 5	1 of 5	20.2	17.8	11.2
Paralog_1-RC _Mk(AV19)	1 of 5	1 of 5	21.4	17.9	16.5
Paralog_2-R _Mk(AV19)	1 of 5	4 of 5	21.6	16.6	13.9
Paralog_2-C _Mk(AV19)	2 of 5	1 of 5	23.7	16.7	14.3

RAPDF = residue-specific all-atom probability discriminatory function (RAPDF) [30]

C-score = internal I-TASSER scoring function [27]

RMSD = all-atom root mean square deviation between the model and the diffraction structure of Hmd enzyme from *M. jannaschii *(PDB ID = 2b0j)

All models are composed of two distinct folding regions, a 200–300 amino acid N-terminal domain which contains both α-helices and β-sheets and a ~50 amino acid C-terminal domain containing only α-helices. According to the diffraction structure of the Hmd enzyme, catalytic activity takes place within the N-terminal domains while dimerization occurs between the C-terminal domains of subunits [8,9]. To gauge the structural conservation between Hmd enzymes and paralogs, root mean square deviations (RMSDs) between the models and the diffraction structure were calculated with respect to the whole protein, the N-terminal domain only, and the C-terminal domain only.

The RMSD between model and diffraction structure is significantly lower with respect to C-terminal domains than N-terminal domains for 10 out of 21 models. These models are Hmd enzyme_RC _from *M. kandleri*, Hmd paralog_1-R_, Hmd paralog_1-C_, and Hmd paralog_2-R _from *M. thermautotrophicus*, Hmd paralog_2-R _and Hmd paralog_2-C _from *M. marburgensis*, Hmd paralog_R _from *M. maripaludis*, Hmd paralog_1-R_, Hmd paralog_2-R_, and Hmd paralog_2-C _from *M. jannaschii*, and Hmd paralog_2-R _and Hmd paralog_2-C _from *M. kandleri*. The RMSD of the C-terminal domains of the Hmd enzyme_RC _from *M. maripaludis *and the diffraction structure of Hmd was higher than that of the N-terminal domain. ClustalW2 multiple sequence alignments [31] of the query protein with its I-TASSER parent structures are available as Additional file 4. Visual analysis of these alignments suggests that the modeling is not biased towards one of the two domains due to sequence similarity with the parent structures. These results therefore indicate that the C-terminal domain is more structurally conserved between Hmd enzyme and paralog than the N-terminal domain.

### Function prediction by Protinfo MFS comparison

The Meta-Functional Signature score (MFS) was used in conjunction with multiple sequence alignment to predict functional sites and functional similarity between Hmd enzymes and paralogs. MFS is part of the Protinfo suite of algorithms  and predicts the functional sites of a protein with higher accuracy than other currently available algorithms [13]. For a given protein, the MFS algorithm quantifies and measures multiple orthogonal features of each amino acid pertaining to either the evolutionary conservation of the amino acid, the contribution of the amino acid to structural integrity, or the frequency in which the residue type itself is found in known functional sites. These features are combined to give the MFS score, which represents the probability that a given amino acid contributes directly to function.

**Figure 4:**
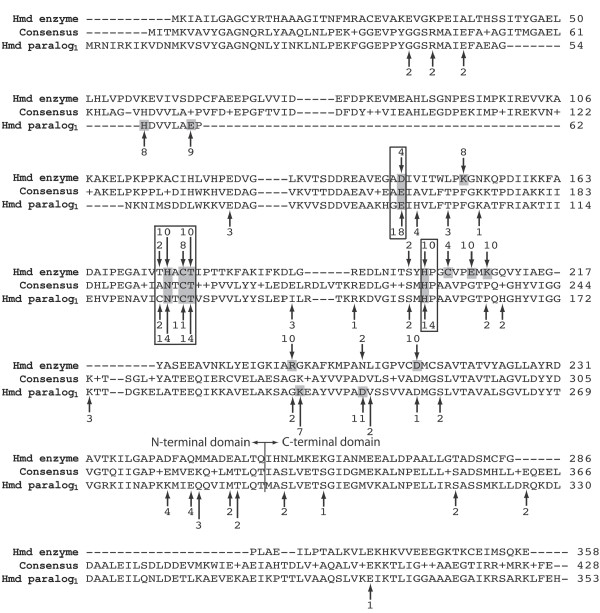
**An abridged multiple sequence alignment with putative functional site tallies**. For clarity, only the consensus sequence along with representative Hmd enzyme and paralog_1 _sequences from *M. jannaschii *are shown. The original multiple sequence alignment containing every sequence is available as Additional file 6. Arrows indicate positions where at least one top ten MFS scoring residue appears in the alignment. Such residues are considered putative functional sites. Arrows referring to putative functional residues from Hmd enzymes are shown above the alignment while those referring to putative functional residues from the Hmd paralogs are shown below the alignment. Numbers at the base of each arrow refer to the quantity of putative functional residues that appear in a single alignment position. Putative functional residues from models that had both the best C-score [27] and RAPDF score [30] are counted twice. Positions in which putative functional residues are found in at least 40% of either Hmd enzymes or Hmd paralogs are highlighted. In five such alignment positions, putative functional sites are found in at least 40% of Hmd enzymes and 40% of Hmd paralogs. Such residue positions are predicted to facilitate a function that is common between Hmd enzymes and paralogs.

MFS scores were calculated for each model summarized in Table 1. The raw MFS data are available as Additional file 5. Any residue with an MFS score in the top ten out of the whole protein was considered a putative functional residue. A ClustalW2 multiple sequence alignment [31] was used to tally the number of putative functional sites that appear in the same alignment position across multiple species (Figure 4). This analysis served two purposes. First, the comparison of putative functional sites across either Hmd enzymes or paralogs provided an *ad hoc *bootstrapping of the MFS predictions. Second, the comparison of putative functional sites between Hmd enzymes and paralogs was used to ascertain whether they share common functional attributes. The unabridged superimposition of MFS data onto a full ClustalW2 alignment of all modeled Hmd proteins is available as Additional file 6.

**Figure 5:**
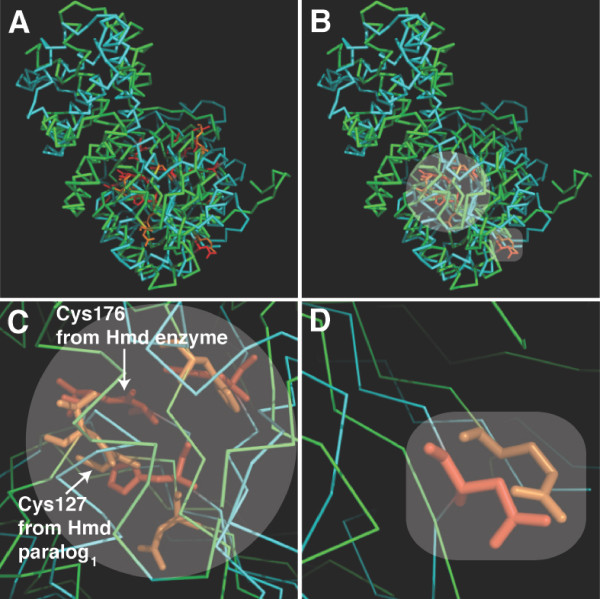
**Predicted functional sites superimposed onto representative tertiary structures of the Hmd enzyme and paralog**. (A) Residues representing alignment positions in either Hmd enzymes (red) or paralogs (orange) with putative functional residues in at least 40% of models are superimposed onto the structures of the Hmd enzyme (blue) and paralog_1 _(green) from *M. jannaschii*. These residues are D143, K151, H174, C176, T177, H201, C204, E207, K209, R235, and D251 in Hmd enzyme from *M. janaschii *and H55, E61, E91, N125, C127, T128, H155, K198, and D206 in Hmd paralog_1 _from *M. janaschii*. (B) Five such residues from each protein are conserved between the Hmd enzymes and paralogs. (C) One cluster contains four such residues from each protein (H174, C176, T177, and H201 from Hmd enzyme and N125, C127, T128, and H154 from Hmd paralog_1_). C176 is known to bind the iron atom and cofactor molecule that coordinate the enzymatic reaction of the Hmd enzyme [8,9]. (D) A second region with no previously characterized functional contribution contains one such residue from each protein (D143 in Hmd enzyme and E94 in Hmd paralog_1_). This figure shows a predicted functional similarity between Hmd enzymes and paralogs and also identifies a putative novel site of common function.

In fifteen such alignment positions, putative functional residues were predicted in at least 40% of either Hmd enzymes or paralogs. In five of these fifteen alignment positions, putative functional sites were predicted in at least 40% of Hmd enzymes and at least 40% of Hmd paralogs. Figure 5A shows representative residues from these fifteen alignment positions mapped onto the diffraction structure of Hmd enzyme [8] and the structure model of Hmd paralog_1 _from *M. jannaschii*. All fifteen residues are located within the N-terminal domain of the protein. The paucity of these residues in the C-terminal domain of either protein is most likely due to its involvement in dimerization rather than enzymatic function.

Four of the five alignment positions in which multiple putative functional residues are conserved between Hmd enzymes and paralogs cluster into a single distinct region (Figure 5B). This cluster is comprised of H174, C176, T177, and H201 in Hmd enzyme and N125, C127, T128, and H154 in Hmd paralog_1 _from *M. jannaschii *(Figure 5C). In the Hmd enzyme from *M. jannaschii*, C176 was previously demonstrated to bind the cofactor and coordinate the iron and substrate [8,9]. This cluster of putative functional sites therefore represents the active site of the Hmd enzyme. The H174 residue of the Hmd enzyme corresponds to the N125 residue of Hmd paralog_1_. Thus the functional importance of this site appears to be conserved while the residue type itself is not. These results are consistent with the independent observations that the Hmd paralog_1 _of *M. jannaschii *is able to competitively bind the Hmd cofactor [10] and that both Hmd paralogs of *M. marburgensis *are unable to catalyze a hydrogenase/dehydrogenase reaction [6] (see Background). A second predicted common functional site between Hmd enzymes and paralogs is comprised of a single amino acid, D143 in Hmd enzyme and E94 in Hmd paralog_1 _(Figure 5D). The functional relevance of this region is yet unknown. There is no experimental evidence that all Hmd paralogs are functionally equivalent. Our analysis however is not dependent on all Hmd paralogs having a single common function. Rather all Hmd paralogs are predicted here to have a common ancestral function and still maintain common features of function, such as the locations of functional sites.

Lipman et al. [11] demonstrated that Hmd paralog_1 _from *M. jannaschii *specifically binds prolyl-tRNA synthetase. The biological significance of this binding has not been examined in a published study since this initial work. Lipman et al. observed that mutations V248A and L252A reduced this binding 4-fold. In our MFS calculation for Hmd paralog_1 _from *M. jannaschii*, V248 has a score of 0.05 and L252 has a score of 0.22. Val and Leu are typically not conserved within protein-protein binding "hot spots" [32]. It may be the case that V248 and L252 represent structurally important residues in Hmd paralog_1 _that do not contribute directly to function. Thus, our MFS analysis cannot confirm the biological relevance of Hmd paralog_1 _binding to prolyl-tRNA synthetase in *M. jannaschii*.

## Conclusion

This study offers an in depth computational analysis of the relationship between the sequences, structures, and functional features of Hmd enzymes and paralogs in class I hydrogenotrophic methanogens. Phylogenetic analysis of thirty *hmd *enzyme and paralog genes from sixteen species and strains confirms that the genetic predecessors of modern Hmd enzymes and paralogs were present in the last common ancestor of all class I hydrogenotrophic methanogens. Structural modeling of fourteen representative Hmd enzymes and paralogs reveals a common structural arrangement comprised of one large N-terminal domain containing α-helices and β-sheets and one smaller C-terminal domain containing only α-helices.

Functional site prediction was performed by the calculation of Meta-Functional Signature (MFS) scores for the fourteen modeled Hmd enzymes and paralogs [13]. MFS comparison across a multiple sequence alignment revealed five functional sites conserved between Hmd enzymes and paralogs. The superimposition of these sites onto representative structures of the Hmd enzyme and paralog showed that the enzyme active site is maintained as a functional site in the paralog. One of the four functionally conserved residues in this functional site is a His in Hmd enzymes and an Asn in most Hmd paralogs. We conclude from these observations that the molecular function of the Hmd paralog is similar but not identical to the enzyme. Our analysis also predicted a second site of common function between Hmd enzymes and paralogs that is yet uncharacterized. Our MFS data did not substantiate the observation of Lipman et al. [11] that Hmd paralog_1 _in *M. jannaschii *specifically binds to prolyl-tRNA synthetase.

Previous experimental work has demonstrated that Hmd paralogs do not enzymatically catalyze hydrogenase/dehydrogenase reactions [6], but are able to competitively bind the Hmd enzyme cofactor [10]. Our results indicate that the catalytic site of the Hmd enzyme is conserved as a functional site in Hmd paralogs, but that the molecular function of the paralog differs from that of the enzyme due to at least one key amino acid substitution. Given these observations, it is possible that the Hmd paralog is responsible for acting as a reservoir for the Hmd enzyme cofactor when H_2 _is low and the Mtd reaction is favored over the Hmd reaction (see Background). Alternatively, the Hmd paralog may act as a scaffold for cofactor synthesis. These hypotheses warrant experimental verification.

The datasets and predictions generated in this study provide a guide for future experimental characterization of the Hmd protein family. This work also serves as an example of detailed protein function prediction that can be achieved by the combination of multiple independent computational techniques. We are currently working to optimize and generalize the method presented here. Such an approach will increase the accuracy of protein function prediction and help to guide the early steps of experimental protein characterization.

## Methods

### Sequence analysis

Thirty Hmd enzyme and paralog sequences from sixteen species and strains were identified using the NCBI implemetation of PSI-BLAST [14] and the multi-genome browser on the MetaCyc server [15]. Three sequences of methanogen prephenate dehydrogenase were also identified and used as an outgroup in the phylogenetic analysis. The boundary between N-terminal and C-terminal domains that is presented in Additional file 1 was ascertained by extrapolating this boundary in the diffraction structure of the Hmd enzyme from *M. jannaschii *across a ClustalW2 multiple sequence alignment [31] of all thirty Hmd sequences. Sequence identities between each pair of proteins were calculated by ClustalW [33]. All of these data are summarized in Additional file 1 along with references and GenInfo Identifiers for each sequence.

### Phylogenetic analysis

Phylogenies were generated separately using the PhyML webserver [24], the Phylip software package [26], and the MrBayes software package [25]. The PhyML phylogeny was calculated using the maximum likelihood method [34] and the WAG substitution matrix [35], which was recommended on the server website. Confidence scores for each branch are bootstrap support values obtained from 500 independent resamplings of alignment positions. The Phylip phylogeny was calculated using the neighbor joining method [36] and the JTT substitution matrix [37]. Confidence scores for each branch are bootstrap support values obtained from 1,000 independent resamplings of alignment positions. The MrBayes phylogenetic trees were calculated using mixed models of amino acid substitution [38], which converged after 10,000 iterations in 100% usage of the WAG substitution model [35]. The MrBayes tree and conditional probability values of the corresponding branches were estimated from 750 tree topologies sampled along 7,500 iterations, following 2,500 burn-in iterations. All three trees were drawn using the Retree and Drawgram programs from the Phylip software package [26]. Trees were relabeled for clarity using graphics editors.

### Structure modeling

Structures were modeled using the I-TASSER webserver, which was determined to be the most accurate structure prediction server in both the CASP7 and CASP8 competitions [12,27,28]. The algorithm threads the query sequence through experimentally solved structures in the SCOP database [29] in order to identify up to five parent structures to be used as comparative modeling templates. Comparative modeling is used to model short segments of the query protein. These segments are then attached by physics-based *de novo *modeling.

I-TASSER returns five models for each amino acid sequence. For all proteins, the most accurate model in each set of five was determined using either the C-score, which is internal to I-TASSER [27], or the residue-specific all-atom probability discriminatory function (RAPDF) [32]. Both of these scoring functions measure the likelihood that a given model is correct with respect to other models of the same protein. C-score is calculated by clustering the thousands of intermediate structures produced during the I-TASSER run. The score is determined by the size of the cluster surrounding each model. RAPDF determines the quality of a model by calculating the sum of logodds scores for all interatomic distances within the model derived from frequencies observed in diffraction structures. The I-TASSER models of Hmd paralog_2 _from *M. jannaschii *had a disconnected main chain. The main chains of these models were made congruent by comparative modeling using the I-TASSER models as templates. This comparative modeling was performed with Protinfo CM [39,40]. Details of all fourteen models are presented in Table 1. Root mean square deviations (RMSDs) of all heavy atoms between the models were calculated using the compare_structures program in the RAMP modeling suite . A concatenated PDB formatted file of the models is available as Additional file 3.

### Function prediction by Protinfo MFS comparison

Meta-Functional Signature (MFS) scores were calculated for each protein using the Protinfo MFS algorithm [13,41]. For a given protein, the MFS algorithm quantifies multiple orthogonal features of each amino acid that pertain to either the evolutionary conservation of the residue, the contribution of the residue to the structural integrity of the protein, or the frequency of the residue type in previously characterized functional sites. These features are combined to produce a score from zero to one that represents the likelihood that the residue is a functional site. Raw MFS data for each modeled protein are available as Additional file 5.

The top ten MFS scoring residues from each protein were considered putative functional sites. A multiple sequence alignment of the corresponding sequences was generated using ClustalW2 [31]. The number of putative functional sites appearing in each alignment position was tallied. Alignment positions in which at least 40% of either Hmd enzymes or paralogs had a putative functional site were identified on representative structures from *M. jannaschii *using the Pymol molecular viewer [42]. An unabridged multiple sequence alignment with highlighted putative functional sites from each protein is available as Additional file 6.

## Authors' contributions

ADG designed this study, performed the analysis, and prepared the majority of this manuscript. JAL and RS contributed to the design of the study and preparation of the manuscript. All authors approved the final manuscript.

## Supplementary Material

Additional file 1 **Sequence Data 1**. A table containing features of the peptide sequences of each gene used in the phylogenetic analysis presented here, their respective GenInfo IDs, and associated references. Click here for file

Additional file 2 **Sequence Data 2**. A ClustalW2 alignment of all Hmd enzyme and paralog peptide sequences used in the phylogenetic analysis presented here. The three prephenate dehydrogenase sequences used as the phylogenetic outgroup are included. Click here for file

Additional file 3 **Protein structure models**. A concatenated file in PDB format of all top C-scoring and RAPDF-scoring protein structure models generated for this study. Click here for file

Additional file 4 **Model-Template Sequence Alignments**. A ClustalW2 alignment between each modeled peptide sequence and the peptide sequences of parent structures used by I-TASSER to create the model. Click here for file

Additional file 5 **MFS Data**. A concatenated file of all raw MFS data generated for this study. Click here for file

Additional file 6 **Function Site Prediction**. All putative functional sites predicted by MFS are highlighted on the full multiple sequence alignment of Hmd enzymes and paralogs. Click here for file

## References

[B1] Bapteste E, Brochier C, Boucher Y (2005). Higher-level classification of the Archaea: evolution of methanogenesis and methanogens. Archaea.

[B2] Gribaldo S, Brochier-Armanet C (2006). The origin and evolution of Archaea: a state of the art. Phil Trans R Soc B.

[B3] Gao B, Gupta RS (2007). Phylogenomic analysis of proteins that are distinctive of Archaea and its main subgroups and the origin of methanogenesis. BMC Genomics.

[B4] Deppenmeier U (2002). The unique biochemistry of methanogenesis. Progr Nucleic Acid Res Mol Biol.

[B5] Reeve JN, Nolling J, Morgan RM, Smith DR (1997). Methanogenesis: genes, genomes and whose on first?. J Bacteriol.

[B6] Afting C, Kremmer E, Brucker C, Hochheimer A, Thauer RK (2000). Regulation of the synthesis of H2-forming methylenetetrahydromethanopterin dehydrogenase (Hmd) and of HmdII and HmdIII in Methanothermobacter marburgensis. Arch Microbiol.

[B7] Hendrickson E, Haydock A, Moore B, Whitman W, Leigh J (2007). Functionally distinct genes regulated by hydrogen limitation and growth rate in methanogenic Archaea. Proc Natl Acad Sci.

[B8] Pilak O, Mamat B, Vogt S, Hagemeier CH, Thaur RK, Shima S, Vonhrein C, Warkentin E, Ermler U (2006). The crystal structure of the apoenzyme of the iron-sulphur cluster-free hydrogenase. J Mol Biol.

[B9] Korbas M, Vogt S, Meyer-Klaucke W, Bill E, Lyon EJ, Thauer RK, Shima S (2006). The iron-sulfur cluster-free hydrogenase (Hmd) is a metalloenzyme with a novel iron binding motif. J Biol Chem.

[B10] Shima S, Thauer RK (2007). A third type of hydrogenase catalyzing H2 activation. Chem Rec.

[B11] Lipman RS, Chen J, Evilia C, Vitseva O, Hou Y-A (2003). Association of an aminoacyl-tRNA synthetase with a putative metabolic protein in archaea. Biochemistry.

[B12] Zhang Y (2007). Template-based modeling and free modeling by I-TASSER in CASP7. Proteins.

[B13] Wang K, Horst J, Cheng G, Nickle D, Samudrala R (2008). Protein meta-functional signatures from combining sequence, structure, evolution and amino acid property information. PLoS Comput Biol.

[B14] Altschul SF, Madden TL, Schaffer AA, Zhang J, Zhang Z, Miller W, Lipman DJ (1997). Gapped BLAST and PSI-BLAST: a new generation of protein database search programs. Nucleic Acids Res.

[B15] Caspi R, Foerster H, Fulcher CA, Kaipa P, Krummenacker M, Latendresse M, Paley S, Rhee SY, Shearer AG, Tissier C, Walk TC, Zhang P, Karp PD (2008). The MetaCyc Database of metabolic pathways and enzymes and the BioCyc collection of Pathway/Genome Databases. Nucleic Acids Res.

[B16] Bult CJ, White O, Olsen GJ, Zhou L, Fleischmann RD, Sutton GG, Blake JA, FitzGerald LM, Clayton RA, Gocayne JD, Kerlavage AR, Dougherty BA, Tomb JF, Adams MD, Reich CI, Overbeek R, Kirkness EF, Weinstock KG, Merrick JM, Glodek A, Scott JL, Geoghagen NS, Venter JC (1996). Complete genome sequence of the methanogenic archaeon, Methanococcus jannaschii. Science.

[B17] Hartmann GC, Klein AR, Linder M, Thauer RK (1996). Purification, properties and primary structure of H2-forming N5,N10-methylenetetrahydromethanopterin dehydrogenase from Methanococcus thermolithotrophicus. Arch Microbiol.

[B18] Hendrickson EL, Kaul R, Zhou Y, Bovee D, Chapman P, Chung J, de Macario EC, Dodsworth JA, Gillett W, Graham DE, Hackett M, Haydock AK, Kang A, Land ML, Levy R, Lie TJ, Major TA, Moore BC, Porat I, Palmeiri A, Rouse G, Saenphimmachak C, Söll D, Van Dien S, Wang T, Whitman WB, Xia Q, Zhang Y, Larimer FW, Olson MV, Leigh JA (2004). Complete Genome Sequence of the Genetically Tractable Hydrogenotrophic Methanogen Methanococcus maripaludis. J Bacteriol.

[B19] Nolling J, Pihl TD, Vriesema A, Reeve JN (1995). Organization and growth phase-dependent transcription of methane genes in two regions of the Methanobacterium thermautotrophicus genome. J Bacteriol.

[B20] Smith DR, Doucette-Stamm LA, Deloughery C, Lee H, Dubois J, Aldredge T, Bashirzadeh R, Blakely D, Cook R, Gilbert K, Harrison D, Hoang L, Keagle P, Lumm W, Pothier B, Qiu D, Spadafora R, Vicaire R, Wang Y, Wierzbowski J, Gibson R, Jiwani N, Caruso A, Bush D, Reeve JN (1997). Complete genome sequence of Methanobacterium thermautotrophicus deltaH: functional analysis and comparative genomics. J Bacteriol.

[B21] Wasserfallen A, Nölling J, Pfister P, Reeve J, de Macario EC (2000). Phylogenetic analysis of 18 thermophilic *Methanobacterium *isolates supports the proposals to create a new genus, *Methanothermobacter *gen. nov., and to reclassify several isolates in three species, *Methanothermobacter thermautotrophicus *comb. nov., *Methanothermobacter wolfeii *comb. nov., and *Methanothermobacter marburgensis *sp. nov. Int J Syst Evol Microbiol.

[B22] Samuel BS, Hansen EE, Manchester JK, Coutinho PM, Henrissat B, Fulton R, Latreille P, Kim K, Wilson RK, Gordon JI (2007). Genomic and metabolic adaptations of Methanobrevibacter smithii to the human gut. Proc Natl Acad Sci.

[B23] Slesarev AI, Mezhevaya KV, Makarova KS, Polushin NN, Shcherbinina OV, Shakhova VV, Belova GI, Aravind L, Natale DA, Rogozin IB, Tatusov RL, Wolf YI, Stetter KO, Malykh AG, Koonin EV, Kozyavkin SA (2002). The complete genome of hyperthermophile Methanopyrus kandleri AV19 and monophyly of archaeal methanogens. Proc Natl Acad Sci.

[B24] Guindon S, Lethiec F, Duroux P, Gascuel O (2005). PHYML Online – a web server for fast maximum likelihood-based phylogenetic inference. Nucleic Acids Res.

[B25] Huelsenbeck J, Ronquist F (2001). MRBAYES: Bayesian inference of phylogenetic trees. Bioinformatics.

[B26] Felsenstein J (1989). PHYLIP – Phylogeny Inference Package (Version 3.2). Cladistics.

[B27] Wu S, Skolnick J, Zhang Y (2007). Ab initio modeling of small proteins by iterative TASSER simulations. BMC Biology.

[B28] Zhang Y (2008). I-TASSER server for protein 3D structure predictions. BMC Bioinformatics.

[B29] Murzin A, Brenner SE, Hubbard T, Chothia C (1995). SCOP: a structural classification of proteins database for the investigation of sequences and structures. J Mol Biol.

[B30] Samudrala R, Moult J (1998). An all-atom distance-dependent conditional probability discriminatory function for protein structure prediction. J Mol Biol.

[B31] Larkin MA, Blackshields G, Brown NP, Chenna R, McGettigan PA, McWilliam H, Valentin F, Wallace IM, Wilm A, Lopez R, Thompson JD, Gibson TJ, Higgins DG (2007). ClustalW and ClustalX version 2. Bioinformatics.

[B32] Ma B, Elkayam T, Wolfson H, Nussinov R (2003). Protein-protein interactions: Structurally conserved residues distinguish between binding sites and exposed protein surfaces. Proc Natl Acad Sci.

[B33] Chenna R, Sugawara H, Koike T, Lopez R, Gibson TJ, Higgins DG, Thompson JD (2003). Multiple sequence alignment with the Clustal series of programs. Nucleic Acids Res.

[B34] Guindon S, Gascuel O (2003). A simple, fast, and accurate algorithm to estimate large phylogenies by maximum likelihood. Syst Biol.

[B35] Whelan S, Goldman N (2001). A general empirical model of protein evolution derived from multiple protein families using a maximum-likelihood approach. Mol Biol Evol.

[B36] Saitou N, Nei M (1987). The neighbor-joining method: a new method for reconstructing phylogenetic trees. Mol Biol Evol.

[B37] Jones DT, Taylor WR, Thornton JM (1992). The Rapid Generation of Mutation Data Matrices from Protein Sequences. Comput Applic Biosci.

[B38] Ronquist F, Huelsenbeck JP (2003). MrBayes 3: Bayesian phylogenetic inference under mixed models. Bioinformatics.

[B39] Hung LH, Ngan SC, Liu T, Samudrala R (2005). PROTINFO: new algorithms for enhanced protein structure predictions. Nucleic Acids Res.

[B40] Hung LH, Samudrala R (2003). PROTINFO: secondary and teritary protein structure prediction. Nucleic Acids Res.

[B41] Wang K, Ram Samudrala (2005). FSSA: A novel method for identifying functional signatures from structural alignments. Bioinformatics.

[B42] DeLano W (2008). The PyMOL Molecular Graphics System.

