# Supplementary material for: Comprehensive computational analysis of Hmd enzymes and paralogs in methanogenic Archaea
Source: BMC Evol Biol. 2009 Aug 11;9:199. doi: 10.1186/1471-2148-9-199 (PMC2739858; doi:10.1186/1471-2148-9-199)
Supplement: Additional file 1 — Sequence Data 1. A table containing features of the peptide sequences of each gene used in the phylogenetic analysis presented here, their respective GenInfo IDs, and associated references. [file 1471-2148-9-199-S1.pdf]

PROTEIN	**E** MJ(2661)	**E** Mtl	**E** Ma(N-3)	**E** Mm(C5)	**E** Mm(S2)	**E** Mv(SB)	**E** Mm(C6)	**E** Mvo(A3)	**E** Mm(C7)	**E** Mvo(PS)
Geninfo ID	15668966	2833439	150401449	134046577	45357690	150399877	159905212	163799431	150403049	2833442
total #aa	299	299	299	299	299	299	299	299	299	299
N-term Domain	1 - 254	1 - 254	1 - 254	1 - 254	1 - 254	1 - 254	1 - 254	1 - 254	1 - 254	1 - 254
C-term Domain	255 - 299	255 - 299	255 - 299	255 - 299	255 - 299	255 - 299	255 - 299	255 - 299	255 - 299	255 - 299
**SEQUENCE SIMILARITIES**										
**E** MJ(2661)	100	88.8	88.7	85.7	85.7	85.9	85.7	88.7	85	87
**E** Mtl	88.8	100	93.4	90.9	90.8	91.1	90.8	91.1	90.1	91.5
**E** Ma(N-3)	88.7	93.4	100	90.4	90.1	90.9	90.1	90.8	89.5	90.5
**E** Mm(C5)	85.7	90.9	90.4	100	97.3	95.8	98.2	92.7	96.6	91.8
**E** Mm(S2)	85.7	90.8	90.1	97.3	100	97	98.3	93.1	97.6	92.4
**E** Mv(SB)	85.9	91.1	90.9	95.8	97	100	96.8	92.4	96	91.4
**E** Mm(C6)	85.7	90.8	90.1	98.2	98.3	96.8	100	93.3	98.2	92.1
**E** Mvo (A3)	88.7	91.1	90.8	92.7	93.1	92.4	93.3	100	91.1	95.6
**E** Mm(C7)	85	90.1	89.5	96.6	97.6	96	98.2	91.1	100	91.3
**E** Mvo(PS)	87	91.5	90.5	91.8	92.4	91.4	92.1	95.6	91.3	100
**E** Mth(w)	82.1	80.1	79.1	80.1	79.8	80.2	80.1	80.6	79.7	80.9
**E** Mth(dH)	82.6	80.8	79.8	79.7	79.7	79.7	79.7	80.6	79.3	81.1
**E** Mtm	82.7	80.8	79.1	79.5	79.7	79.9	79.5	80.3	79.1	80.8
**E** Ml(Z)	81.6	80.8	79.1	78.4	78.8	78.4	78.5	79.1	78.7	80.1
**E** Ms(ATCC)	82.3	80.4	80.7	81.3	81.3	81.5	81.5	82	81.5	81.9
**E** Mk(AV19)	79.5	78.6	78.1	76.6	76.6	76.1	76.7	77.1	76.4	78.1
**P2** Mj(2661)	60.5	60.1	60.1	60.7	60.8	59	60.7	61.1	58.9	59.6
**P** Ma(N-3)	59.6	59.6	59.4	58.6	58.5	58.9	58.6	58.7	58.9	58.9
**P** Mv(SB)	60.1	60.9	59.2	59.1	58.9	59.7	58.9	59.5	59.7	59.1
**P1** Mj(2661)	60.2	60.2	59.5	59.3	59.3	59.8	59.5	59.2	59.5	59.6
**P1** Mth(dH)	59.1	58.9	59.1	58.5	58.8	59.4	58.8	58.7	58.9	59.5
**P2** Mth(dH)	59.7	61.8	60.1	59.2	59.3	60	59.4	61.1	59.3	61.8
**P1** Mtm	59.7	60	60.1	58.9	58.9	59.4	59	58.7	59	60.8
**P2** Mtm	59.6	61.6	60.8	59	59.2	59.6	59.3	60.6	59.2	61.4
**P** Mm(C7)	60.1	60.1	59.5	58.9	58.8	58.8	58.9	58.9	58.9	58.9
**P** Mm(C6)	60.1	60.1	59.5	58.8	58.7	58.8	58.9	58.9	58.9	58.9
**P** Mm(S2)	60.1	60.7	59.2	58.8	58.7	59.2	58.8	58.7	58.8	58.7
**P** Mm(C5)	59.8	59.8	59.2	58.7	58.5	58.5	58.8	58.7	58.8	58.7
**P1** Mk(AV19)	57.4	58.6	58	56.6	56.9	57.5	56.6	58	56.6	58
**P2** Mk(AV19)	59.3	58.5	58.6	58.2	58.3	58.8	58.5	58.6	58.8	58.8
**Pd** Ma(N-3)	52.8	54.5	55.3	53.8	52.1	53.2	52.1	54.8	52.1	55.2
**Pd** Mj(2661)	53.6	54.5	53.8	55.1	52.1	53.1	53.7	54.1	54.7	54.5
**Pd** Mm(C7)	55.7	53.5	56.1	53.1	53.5	53.4	53.4	54.8	53.4	56.6

PROTEIN	**E** Mth(dH)	**E** Mtm	**E** Ml(Z)	**E** Ms(ATCC)	**E** Mk(AV19)	**P2** Mj(2661)	**P** Ma(N-3)	**P** Mv(SB)	**P1** Mj(2661)
Geninfo ID	15679153	2828203	124486556	148642632	20093453	15668897	150401123	150399766	15669529
total #aa	304	301	298	295	310	250	250	250	261
N-term Domain	1 - 259	1 - 256	1 - 253	1 - 250	1 - 265	1 - 205	1 - 205	1 - 205	1 - 216
C-term Domain	260 - 304	257 - 301	254 - 298	251 - 295	266 - 310	206 - 250	206 - 250	206 - 250	217 - 261
**SEQUENCE SIMILARITIES**									
**E** MJ(2661)	82.6	82.7	81.6	82.3	79.5	60.5	59.6	60.1	60.2
**E** Mtl	80.8	80.8	80.8	80.4	78.6	60.1	59.6	60.9	60.2
**E** Ma(N-3)	79.8	79.1	79.1	80.7	78.1	60.1	59.4	59.2	59.5
**E** Mm(C5)	79.7	79.5	78.4	81.3	76.6	60.7	58.6	59.1	59.3
**E** Mm(S2)	79.7	79.7	78.8	81.3	76.6	60.8	58.5	58.9	59.3
**E** Mv(SB)	79.7	79.9	78.4	81.5	76.1	59	58.9	59.7	59.8
**E** Mm(C6)	79.7	79.5	78.5	81.5	76.7	60.7	58.6	58.9	59.5
**E** Mvo (A3)	80.6	80.3	79.1	82	77.1	61.1	58.7	59.5	59.2
**E** Mm(C7)	79.3	79.1	78.7	81.5	76.4	58.9	58.9	59.7	59.5
**E** Mvo(PS)	81.1	80.8	80.1	81.9	78.1	59.6	58.9	59.1	59.6
**E** Mth(w)	98.5	97.7	87.7	85.8	78.1	59.5	59	59.1	58.9
**E** Mth(dH)	100	98.4	87.4	85.8	77.5	59.8	59	59.5	59.2
**E** Mtm	98.4	100	86.1	85.3	77.8	59.9	58.6	59.2	59
**E** Ml(Z)	87.4	86.1	100	83.1	76.2	61.1	58.3	59.5	60.1
**E** Ms(ATCC)	85.8	85.3	83.1	100	79.8	59.2	58.8	58.8	59.3
**E** Mk(AV19)	77.5	77.8	76.2	79.8	100	60.7	59.8	59.7	58.6
**P2** Mj(2661)	59.8	59.9	61.1	59.2	60.7	100	78.6	77.8	82.1
**P** Ma(N-3)	59	58.6	58.3	58.8	59.8	78.6	100	87.4	85.9
**P** Mv(SB)	59.5	59.2	59.5	58.8	59.7	77.8	87.4	100	85.5
**P1** Mj(2661)	59.2	59	60.1	59.3	58.6	82.1	85.9	85.5	100
**P1** Mth(dH)	59.9	58.3	59.6	58.9	59.5	76	81.3	81.2	81.6
**P2** Mth(dH)	59.2	59	61.1	59.3	59.2	78.6	83.1	83.4	84.6
**P1** Mtm	60.3	59	58.5	59.5	60.3	76.2	80.3	81.2	81.5
**P2** Mtm	59.2	59.2	60.1	59.2	59.6	77.1	82.2	82.7	83.7
**P** Mm(C7)	59.7	59.5	59.2	59.1	61.3	77.4	87.1	92.1	86.2
**P** Mm(C6)	59.7	59.5	58.9	58.8	60.1	76.9	88.1	93.1	86.1
**P** Mm(S2)	60.1	59.1	59.7	58.9	60.9	77.1	87.7	93.1	85.6
**P** Mm(C5)	59.7	59.5	58.9	58.6	61.1	76.8	87.7	92.5	85.5
**P1** Mk(AV19)	60.7	60.8	58.6	58.5	58.1	66.2	66.8	65.6	68.1
**P2** Mk(AV19)	59.4	59.3	59.3	57.9	60.2	65.6	66.8	64.1	67.7
**Pd** Ma(N-3)	53	53.1	54.2	53.9	52.4	57.1	58.7	58.5	57.6
**Pd** Mj(2661)	53.6	53.3	53.9	53.9	53.6	55.3	56.1	56.3	55.9
**Pd** Mm(C7)	51.6	53.1	55.1	53.3	56	56.7	56.9	55.1	57.5

**PROTEIN**	**P1** Mth(dH)	**P2** Mth(dH)	**P1** Mtm	**P2** Mtm	**P** Mm(C7)	**P** Mm(C6)	**P** Mm(S2)	**P** Mm(C5)	**P1** Mk(AV19)
Geninfo ID	15678532	15679509	7573569	7573567	150402915	159905340	45359279	134046716	20094558
total #aa	256	253	253	253	250	250	250	250	228
N-term Domain	1 - 211	1 - 208	1 - 208	1 - 208	1 - 205	1 - 205	1 - 205	1 - 205	1 - 184
C-term Domain	212 - 256	209 - 253	209 - 253	209 - 253	206 - 250	206 - 250	206 - 250	206 - 250	185 - 228
**SEQUENCE SIMILARITIES**									
**E** MJ(2661)	59.1	59.7	59.7	59.6	60.1	60.1	60.1	59.8	57.4
**E** Mtl	58.9	61.8	60	61.6	60.1	60.1	60.7	59.8	58.6
**E** Ma(N-3)	59.1	60.1	60.1	60.8	59.5	59.5	59.2	59.2	58
**E** Mm(C5)	58.5	59.2	58.9	59	58.9	58.8	58.8	58.7	56.6
**E** Mm(S2)	58.8	59.3	58.9	59.2	58.8	58.7	58.7	58.5	56.9
**E** Mv(SB)	59.4	60	59.4	59.6	58.8	58.8	59.2	58.5	57.5
**E** Mm(C6)	58.8	59.4	59	59.3	58.9	58.9	58.8	58.8	56.6
**E** Mvo (A3)	58.7	61.1	58.7	60.6	58.9	58.9	58.7	58.7	58
**E** Mm(C7)	58.9	59.3	59	59.2	58.9	58.9	58.8	58.8	56.6
**E** Mvo(PS)	59.5	61.8	60.8	61.4	58.9	58.9	58.7	58.7	58
**E** Mth(w)	58.4	59	59.2	59	59.2	59.2	59.7	59.2	60.8
**E** Mth(dH)	59.9	59.2	60.3	59.2	59.7	59.7	60.1	59.7	60.7
**E** Mtm	58.3	59	59	59.2	59.5	59.5	59.1	59.5	60.8
**E** Ml(Z)	59.6	61.1	58.5	60.1	59.2	58.9	59.7	58.9	58.6
**E** Ms(ATCC)	58.9	59.3	59.5	59.2	59.1	58.8	58.9	58.6	58.5
**E** Mk(AV19)	59.5	59.2	60.3	59.6	61.3	60.1	60.9	61.1	58.1
**P2** Mj(2661)	76	78.6	76.2	77.1	77.4	76.9	77.1	76.8	66.2
**P** Ma(N-3)	81.3	83.1	80.3	82.2	87.1	88.1	87.7	87.7	66.8
**P** Mv(SB)	81.2	83.4	81.2	82.7	92.1	93.1	93.1	92.5	65.6
**P1** Mj(2661)	81.6	84.6	81.5	83.7	86.2	86.1	85.6	85.5	68.1
**P1** Mth(dH)	100	88.8	94.6	88.5	83.4	83.6	82.1	83.7	65.4
**P2** Mth(dH)	88.8	100	89.2	97.7	83.4	83.6	82.1	83.7	65.4
**P1** Mtm	94.6	89.2	100	88.8	81.7	81.5	81.4	81.1	66.7
**P2** Mtm	88.5	97.7	88.8	100	82.1	82.1	82.6	83.1	66
**P** Mm(C7)	83.4	83.4	81.7	82.1	100	98.5	97.4	98.2	66.4
**P** Mm(C6)	83.6	83.6	81.5	82.1	98.5	100	96.8	97.9	66.4
**P** Mm(S2)	82.1	82.1	81.4	82.6	97.4	96.8	100	95.9	65.7
**P** Mm(C5)	83.7	83.7	81.1	83.1	98.2	97.9	95.9	100	65.9
**P1** Mk(AV19)	65.4	65.4	66.7	66	66.4	66.4	65.7	65.9	100
**P2** Mk(AV19)	66.1	66.5	66.8	66.7	65.7	65.9	65.1	65.4	67.3
**Pd** Ma(N-3)	55.8	55.8	55.7	56.5	58.2	58.4	58.1	57.5	55.8
**Pd** Mj(2661)	56.3	56.3	57.1	55.1	55.9	55.6	54.1	56.5	56.4
**Pd** Mm(C7)	55.1	55.1	55.5	54.9	56.7	57.3	56.9	57	58

**PROTEIN**	**P2** Mk(AV19)	**Pd** Ma(N-3)	**Pd** Mj(2661)	**Pd** Mm(C7)
Geninfo ID	20094801	150401448	15668793	150402684
total #aa	248	342	346	344
N-term Domain	1 - 204	1 - 297	1 - 301	1 - 299
C-term Domain	205 - 248	297 - 342	302 - 346	300 - 344
**SEQUENCE SIMILARITIES**				
**E** MJ(2661)	59.3	52.8	53.6	55.7
**E** Mtl	58.5	54.5	54.5	53.5
**E** Ma(N-3)	58.6	55.3	53.8	56.1
**E** Mm(C5)	58.2	53.8	55.1	53.1
**E** Mm(S2)	58.3	52.1	52.1	53.5
**E** Mv(SB)	58.8	53.2	53.1	53.4
**E** Mm(C6)	58.5	52.1	53.7	53.4
**E** Mvo (A3)	58.6	54.8	54.1	54.8
**E** Mm(C7)	58.8	52.1	54.7	53.4
**E** Mvo(PS)	58.8	55.2	54.5	56.6
**E** Mth(w)	59.6	52.8	53.5	51.6
**E** Mth(dH)	59.4	53	53.6	51.6
**E** Mtm	59.3	53.1	53.3	53.1
**E** Ml(Z)	59.3	54.2	53.9	55.1
**E** Ms(ATCC)	57.9	53.9	53.9	53.3
**E** Mk(AV19)	60.2	52.4	53.6	56
**P2** Mj(2661)	65.6	57.1	55.3	56.7
**P** Ma(N-3)	66.8	58.7	56.1	56.9
**P** Mv(SB)	64.1	58.5	56.3	55.1
**P1** Mj(2661)	67.7	57.6	55.9	57.5
**P1** Mth(dH)	66.1	55.8	56.3	55.1
**P2** Mth(dH)	66.5	55.8	56.3	55.1
**P1** Mtm	66.8	55.7	57.1	55.5
**P2** Mtm	66.7	56.5	55.1	54.9
**P** Mm(C7)	65.7	58.2	55.9	56.7
**P** Mm(C6)	65.9	58.4	55.6	57.3
**P** Mm(S2)	65.1	58.1	54.1	56.9
**P** Mm(C5)	65.4	57.5	56.5	57
**P1** Mk(AV19)	67.3	55.8	56.4	58
**P2** Mk(AV19)	100	53.7	56.8	52.9
**Pd** Ma(N-3)	53.7	100	80.9	81.3
**Pd** Mj(2661)	56.8	80.9	100	81.8
**Pd** Mm(C7)	52.9	81.3	81.8	100

Genes:

E =	H2-forming 4(H)MPT dehydrogenase
P =	H2-forming 4(H)MPT dehydrogenase paralog
Pd =	Prephenate dehydrogenase

Organisms:

MJ(2661) =	Methanocaldococcus jannaschii strain 2661
Mtl =	Methanococcus thermolithotrophicus
Ma (N-3) =	Methanococcus aeolicus Nankai-3
Mm(C5) =	Methanococcus maripaludis strain C5
Mm(S2) =	Methanococcus maripaludis strain S2
Mv(SB) =	Methanococcus vannielii strain SB
Mm(C6) =	Methanococcus maripaludis strain C6
Mvo (PS) =	Methanococcus voltae strain PS
Mvo (A3) =	Methanococcus voltae strain A3
Mm(C7) =	Methanococcus maripaludis strain C7
Mth(w) =	Methanothemrobacter thermautotrophicus strain Winter
Mth(dH) =	Methanothermobacter thermautotrophicus strain delta H
Mtm =	Methanothermobacter marburgensis
Ml(Z) =	Methanocorpusculum labreanum strain Z
Ms(ATCC) =	Methanobrevibacter smithii ATCC 35061
Mk(AV19) =	Methanopyrus kandleri AV19

References:

MJ(2661) =	[16]
Mtl =	[17]
Ma (N-3) =	Copeland, A, *et al.*: **Complete sequence of Methanococcus aeolicus Nankai-3**. *US DOE Joint Genome Institute* (unpublished).
Mm(C5) =	Copeland, A, *et al.*: **Complete sequence of chromosome of Methanococcus maripaludis C5**. *US DOE Joint Genome Institute* (unpublished).
Mm(S2) =	[18]
Mv(SB) =	Copeland, A, *et al.*: **Complete sequence of Methanococcus vannielii SB**. *US DOE Joint Genome Institute* (unpublished).
Mm(C6) =	Copeland, A, *et al.*: **Complete sequence of Methanococcus maripaludis C6**. *US DOE Joint Genome Institute* (unpublished).
Mvo (PS) =	Hartmann G, Thauer RK: **Hmd genomic sequence**. *Direct Submission to NCBI databases* 1996.
Mvo (A3) =	Copeland, A, *et al.*: **Sequencing of the draft genome and assembly of Methanococcus voltae A3**. *US DOE Joint Genome Institute (JGI-PGF)* (unpublished).
Mm(C7) =	Copeland, A, *et al.*: **Complete sequence of Methanococcus maripaludis C7**. *US DOE Joint Genome Institute* (unpublished).
Mth(w) =	[19]
Mth(dH) =	[20]
Mtm =	[21]
Ml(Z) =	Copeland, A, *et al.*: **Complete sequence of Methanocorpusculum labreanum Z**. *US DOE Joint Genome Institute* (unpublished).
Ms(ATCC) =	[22]
Mk(AV19) =	[23]
