# Supplementary material for: Comprehensive computational analysis of Hmd enzymes and paralogs in methanogenic Archaea
Source: BMC Evol Biol. 2009 Aug 11;9:199. doi: 10.1186/1471-2148-9-199 (PMC2739858; doi:10.1186/1471-2148-9-199)
Supplement: Additional file 6 — Function Site Prediction. All putative functional sites predicted by MFS are highlighted on the full multiple sequence alignment of Hmd enzymes and paralogs. [file 1471-2148-9-199-S6.pdf]

```
Hmd E Mm(S2)          ------------MKVAILGAGCYRTHAASGITNFSRASQVAKEAGIPEIAMTHSTITMGAE 49
Hmd E Mj(DSM_2661)    ------------MKIAILGAGCYRTHAAAGITNFMRACEVAKEVGKPEIALTHSSITYGAE 49
Hmd E Mth(dH)         ---------MIDMKLAILGAGCYRTHAASGITNFSRACEVAEMVGKPEIAMTHSTITMGAE 52
Hmd E Mtm             ------------MKLAILGAGCYRTHAASGITNFSRACEVAEMVGKPEIAMTHSTITMGAE 49
Hmd E Mk(AV19)        --------MVEINKVAILGAGCWRTHAATGITTFKRACEVADETGIKEAALTHSSVTYAVE 53
Hmd P Mm(S2)          -----------MKVTVYGAGNQNLYVNQLNLPGTYGGVPPYGGSRMAIEFAKAG------ 43
Hmd P1 Mj(DSM_2661)   MRNIRKIKVDNMKVSVYGAGNQNLYINKLNLPEKFGGEPPYGGSRMAIEFAEAG------ 54
Hmd P2 Mj(DSM_2661)   -----------MKISIYGAGNQKLYLEQLKVPEKFGGEPPYGGAGMAIEFAKAG------ 43
Hmd P1 Mt(dH)         -----MKRVIAMKVTVYGAGNQKLYTDELNLPERYGGEAPYGGSRMAMEFAMAG------ 49
Hmd P1 Mtm            --------MITMKVTVYGAGNQKLYTEELNLPEKYGGEAPYGGSRMAMEFAQAG------ 46
Hmd P2 Mt(dH)         --------MSTMKVAVYGAGNQKLYVDQLNLPEKYGGEAPYGGSRMAIEFAEAG------ 46
Hmd P2 Mtm            --------MSTMKVAVYGAGNQKLYVDQLNLPEKYGGEAPYGGSRMAIEFAEAG------ 46
Hmd P1 Mk(AV19)       -------------MATVAVLGFGNQRLYERIG-AAEKLGGEPPFGGAAMAIEFAEAG------ 43
Hmd P2 Mk(AV19)       --------------------------------MEVHYELPLGGAHLAYRAAEAG------ 22

Hmd E Mm(S2)          LLHLIPEITEVVVSDPCFAEEPGMVVLD-----QFDYKAVMEAHLAGDAEKVMPEIREAV 104
Hmd E Mj(DSM_2661)    LLHLVPDVKEVIVSDPCFAEEPGLVVID-----EFDPKEVMEAHLSGNPESIMPKIREVV 104
Hmd E Mth(dH)         LKELAG-VDEVVVADPVFDN--QFTVID-----DFAYEDVIEAHKE-DPEKIMPQIREKV 103
Hmd E Mtm             LKELAG-VDEVVVADPVFDN--QFTVID-----DFAYEDVIEAHKE-DPENIMPQIREKV 100
Hmd E Mk(AV19)        LKHLAG-VDEVVLSDPVFDAD-GFTVVDIEEDCDVDLDEFIKAHLEGNPEDVMPKLRDYV 111
Hmd P Mm(S2)          --------HDVILAEV-------------------------------------------- 51
Hmd P1 Mj(DSM_2661)   --------HDVVLAEP-------------------------------------------- 62
Hmd P2 Mj(DSM_2661)   --------HDVVLSEP-------------------------------------------- 51
Hmd P1 Mt(dH)         --------HDVILAEP-------------------------------------------- 57
Hmd P1 Mtm            --------HEVTLAEP-------------------------------------------- 54
Hmd P2 Mt(dH)         --------HDVVLAEP-------------------------------------------- 54
Hmd P2 Mtm            --------HDVVLAEP-------------------------------------------- 54
Hmd P1 Mk(AV19)       --------HDVVLADP-------------------------------------------- 51
Hmd P2 Mk(AV19)       --------HDVVLCDP-------------------------------------------- 30

Hmd E Mm(S2)          KAKAKETPKPPKGCIHFVHPETVG----LKVTASDVEAVKDADIVITWLPKGGSQPAIIE 160
Hmd E Mj(DSM_2661)    KAKAKELPKPPKACIHLVHPEDVG----LKVTSDDREAVEGADIVITWLPKGNKQPDIIK 160
Hmd E Mth(dH)         NEVAKELPKPPEGAIHFTHPEDLG----FEITTDDREAVADADFIMTWFPKGDMQPDIID 159
Hmd E Mtm             NEVAKELPKPPEGAIHFTHPEDLG----FEITTDDREAVADADFIMTWFPKGDMQPDIIN 156
Hmd E Mk(AV19)        NDIADDVPKPPKGAIHFLSPEEMEDKLDIVVTTDDAEAVEDADMIISWLPKGGVQPDIFK 171
Hmd P Mm(S2)          -------NKSNLTDEQWKIVEEAG----VKVLTDDAEAAKEAEIAIFFTPFGKKTVEIAK 100
Hmd P1 Mj(DSM_2661)   -------NKNIMSDDLWKKVEDAG----VKVVSDDVEAAKHGEIHVLFTPFGKATFRIAK 111
Hmd P2 Mj(DSM_2661)   -------NRDVMSDDLWKKVEDAG----VKVVSDDIEAAKHGEIHVLFTPFGRITLNIAN 100
Hmd P1 Mt(dH)         -------SREFLDDEQWSMVEEAG----VTVTDNDKEAASEAEVAVLFTPFGKGTFSIAR 106
Hmd P1 Mtm            -------VRDVLEDGHWNLVEEAG----VTVTDNDKEASAEADVAVLFTPFGKGTVRIAR 103
Hmd P2 Mt(dH)         -------ARDMLDDAHWKVVEDAG----VTVTDNDAEAASEAEIAVLFTPFGKKTFEIAK 103
Hmd P2 Mtm            -------AREMLDDAHWKVVEDAG----VTVTDSDAEAASEAEMAVLFTPFGKKTFDIAK 103
Hmd P1 Mk(AV19)       -------NLSEQDPEHVDRVADAG----VELTEDDAQAVEGAEMVVLFTPFG-ATGGIIR 99
Hmd P2 Mk(AV19)       -------SFDISE-ESMERLESAG----VELVTDDTAGVEHGEIQILFTPFG-HTVKIAR 77

Hmd E Mm(S2)          KFASEIKKGAIVTHACTIPTPKFAKIFKDLG------RDDLNIIAYHPGAVPEMKGQAFL 214
Hmd E Mj(DSM_2661)    KFADAIPEGAIVTHACTIPTTKFAKIFKDLG------REDLNITSYHPGCVPEMKGQVYI 214
Hmd E Mth(dH)         KFIDDIKPGAIVTHACTIPTTKFYKIFEEKHGDLVTKPETLNVTSYHPGAVPEMKGQVYI 219
Hmd E Mtm             KFIDDIKPGAIVTHACTIPTTKFYKIFEQKHGDLVTKPETLNVTSYHPGAVPEMKGQVYI 216
Hmd E Mk(AV19)        KIIDDIPEGCIVANTCTIPTTRQFKEMFEDMG------RDDLQVTSYHPATVPEHKGQVFV 225
Hmd P Mm(S2)          TILPHLPQNAIIANTCTVSPVVLIYTMLEVELR---TKRKDIGITSMHPAAVPGTPQHGHY 157
Hmd P1 Mj(DSM_2661)   TIIEHVPENAVICNTCTVSPVVLYYSLEPILR---TKRKDVGISSMHPAAVPGTPQHGHY 168
Hmd P2 Mj(DSM_2661)   TIIEHVPENAIICNTCTIPTPVLYRSLEGILR---LKRRDVGISSMHPTGVPGTPSQKYY 157
Hmd P1 Mt(dH)         EILPELPEGSVIANTCTVSPVVLYYVLEKELK---LERTDVGVSSMHPAAVPGTPQHGHY 163
Hmd P1 Mtm            EILPELPEGAVIANTCTVSPVVLYYVLEKELK---MDRTDIGVSSMHPAAVPGTPQHEHY 160
Hmd P2 Mt(dH)         NITNHLPEGAVIANTCTVSPVVLYYVLERELR---RDRKDLGIASMHPAAVPGTPQHGHY 160
Hmd P2 Mtm            DITKHLPEGAVIANTCTVSPVVLYYVLERELR---RDRQDLGIASMHPAAVPGTPQHGHY 160
Hmd P1 Mk(AV19)       EIASHLEEGAVVCPTCTSSAFEIHESLYEAGL---EVPEAVGVMPAHPAGIPGTENHRAY 156
Hmd P2 Mk(AV19)       ELLDDVREGAVLCTTCTCPPIALYHGLEGELR---TKREDVGVSSFHPAGIPGAETQDLI 134

Hmd E Mm(S2)          SEG----------LADAEKVEEFYCMAKTARGEAFKMPANLISPVCDMGSAVTAPVYAAI 264
Hmd E Mj(DSM_2661)    AEG----------YASEEAVNKLYEIGKIARGKAFKMPANLIGPVCDMCSAVTATVYAGL 264
Hmd E Mth(dH)         AEG----------YASEEAINTLFELGQKARGNAYRLPAELLGPVCDMCSALTAITYAGI 269
Hmd E Mtm             AEG----------YASEDAIETLFELGQKARGNAYRLPAELLGPVCDMCSALTAMTYACI 266
Hmd E Mk(AV19)        AEG----------YADEEVVEAVYELGEKARGLAFKVPGYLLGPVCDMASAVTAIVYAGL 275
Hmd P Mm(S2)          VISSKAT--NGTEYASDEQIEKCVKLTESIEKTPYLVPADVSATVSDMGSLLTAVTLAGV 215
Hmd P1 Mj(DSM_2661)   VIGGKTT--DGKELATEEQIKKAVELAKSAGKEAYVVPADVSSVVADMGSLVTAVALSGV 226
Hmd P2 Mj(DSM_2661)   TIAGKAL--EGKEYATEDQINKLVELVKSVGKIPYVTPADVPAVVADMGALVTAVALVGV 215
Hmd P1 Mt(dH)         VIGGRPT--AELDMATDEQVSRCAELAESTGKKAYIVPADVTSAVADMGSLVTAVALAGV 221
Hmd P1 Mtm            VIGGRPT--AELDIATDEQIARCAELAESTGKTAYVVPADVTSAVADMGSLVTAVALAGV 218
Hmd P2 Mt(dH)         VIGGHSS--SELDIATDEQISRCVELAESCGKVAYVVPADVSSAVADMGSLVTAVTLSGV 218
Hmd P2 Mtm            VIGGHSS--SELDIATDEQISRCVELAESCGKTAYVVPADVSSAVADMGSLVTAVTLSGV 218
Hmd P1 Mk(AV19)       ITARGTG--NGTVLATEEQAELVEEVLSSTGKEVFELPHVELVSVVGDLSVVLLKRVIEA 214
Hmd P2 Mk(AV19)       LVADARSEESGIELASEEQVERCVNLAEDMGYDVVYVL-DPELISSIGDMSVVLTVRIVQA 193

                       N-terminal domain <---|---> C-terminal domain
Hmd E Mm(S2)          LAYRDAVTQILGAPADFAQMMADEAISQILDLMRNEGIKNMEDKLNPKALTGTADSMCFG 324
Hmd E Mj(DSM_2661)    LAYRDAVTKILGAPADFAQMMADEALTQIHNLMKEKGIANMEEALDPAALLGTADSMCFG 324
Hmd E Mth(dH)         LSYRDSVTQVLGAPAGFAQMMAKESLEQITALMEKVGIDKMEENLDPGALLGTADSMNFG 329
Hmd E Mtm             LSYRDSVTQVLGAPASFAQMMAKESLEQITALMEKVGIDKMEENLDPGALLGTADSMNFG 326
Hmd E Mk(AV19)        LTFRDACTDILGAPVDFTQNMAVEALQMMAKFMEEEGLDKLEEALDPAALTNTADSMNFG 335
Hmd P Mm(S2)          LDYYSVGTKVIKAPKKMVEQQILMTLQTMASLVESSGVDGLVKALNPELLSKAASSMHLL 275
Hmd P1 Mj(DSM_2661)   LDYYTVGRKIINAPKKMIEQQVIMTLQTMASLVETSGIEGMVKALNPELLIRSASSMKLL 286
Hmd P2 Mj(DSM_2661)   LDYYRVGTQIINAPKDMIEKQILISLQTIASIIETSGMEGLMKVFNKDALLSSAKNMLID 275
Hmd P1 Mt(dH)         LDYYYVGTQIIKAPEEMVEKQILMTLQTMASLVETSGVDGMAAAINPELLVKSARSMHLL 281
Hmd P1 Mtm            LDYYYVGTQIIKAPEEMVEKQILMTLQTMASLVETSGVDGMARAMNPELLVKSAKSMHLL 278
Hmd P2 Mt(dH)         LDYYYVGTQIIRAPKEMVEKQILMTLQTIASLVETSGVNGMLKAMNPELLVRSAKSMHLL 278
Hmd P2 Mtm            LDYYYVGTQIIKAPVEMVEKQILMTLQTIASLVETSGVNGMLKAMNPELLVRSAKSMHLL 278
Hmd P1 Mk(AV19)       LKEFCAVKALG-APQEMIDRQAMMTLATLAALIEAGGIGG---------LLETLDEEAIE 264
Hmd P2 Mk(AV19)       ICNYFSVARICGAPIEMIEEQVEEVLSTMAYLVGRYGLDAFDRMD-GKLLLRSLRNMALT 252

Hmd E Mm(S2)          ---------------------------PLAD--ILPASLKVLEKH-ANENKCECGCSIKP 354
Hmd E Mj(DSM_2661)    ---------------------------PLAE--ILPTALKVLEKHKVVEEGKTKCEIMS 355
Hmd E Mth(dH)         ---------------------------ASAD--ILPTVFEILEKRKK------------- 347
Hmd E Mtm             ---------------------------ASAE--ILPTVFEILEKRKK------------- 344
Hmd E Mk(AV19)        ---------------------------PLADTEILPKALEVLEKYSKKAE---------- 358
Hmd P Mm(S2)          DQQKDLDAALEILTHLDGELEKASTVAEIKPTTLVAAQSLVKELETLIGGAAANGAIKRS 335
Hmd P1 Mj(DSM_2661)   DRQKDLDAALEILQNLDETLKAEVEKAEIKPTTLVAAQSLVKEIKTLIGGAAAEGAIKRS 346
Hmd P2 Mj(DSM_2661)   ERQEDLNLALKIIEEFDKST---IGEKDISQTYLVAPQALIKEAVSLIGKSAVEGMIRRS 332
Hmd P1 Mt(dH)         DEQEELDAALKKISDLNDDVMKWIEGAEVNHTDLVAAQQALTGELKTVIGDRAAEGTIRRC 341
Hmd P1 Mtm            DEQEELDAALRKISQLNDDVMKWIEGAEVKHTDLVAAQQALTGELKTVMGDRAAEGTIRRC 338
Hmd P2 Mt(dH)         EEQEELDAALNTLSDLDDEVNKWIEKGEIRHTDLVAAQQALAKEIKNLMGGKAAEGTIRRC 338
Hmd P2 Mtm            EEQEELDAAINTLADLDDEVTQWINKGEIRHTDLVAAQQALAKELKNLMGGKAAEGTIRRC 338
Hmd P1 Mk(AV19)       ASYSNMEPFVDG--------VEEPEGEPVERFVVLPGEATREAVIELVGERGWRTVRMRA 316
Hmd P2 Mk(AV19)       EDVERVVDFFERC--YVDLLSGDRESMRELSSMMVPPDKLVDRVREIVGEAPVQYARRRF 310

Hmd E Mm(S2)          --------
Hmd E Mj(DSM_2661)    QKE-----  358
Hmd E Mth(dH)         --------
Hmd E Mtm             --------
Hmd E Mk(AV19)        --------
Hmd P Mm(S2)          TKKLFQ--  341
Hmd P1 Mj(DSM_2661)   ARKLFEH-  353
Hmd P2 Mj(DSM_2661)   SNKLFK--  338
Hmd P1 Mt(dH)         MRKMFE--  347
Hmd P1 Mtm            MRKMFE--  344
Hmd P2 Mt(dH)         MRKMFE--  344
Hmd P2 Mtm            MRKMFE--  344
Hmd P1 Mk(AV19)       WVDLYKKH  324
Hmd P2 Mk(AV19)       FEERRWGS  318
```

= top 10 MFS scoring residue in best RAPDF model
= top 10 MFS scoring residue in best C-score model
= top 10 MFS scoring residue in both models
